# Tumor Budding, Micropapillary Pattern, and Polyploidy Giant Cancer Cells in Colorectal Cancer: Current Status and Future Prospects

**DOI:** 10.1155/2016/4810734

**Published:** 2016-10-23

**Authors:** Shiwu Zhang, Dan Zhang, Zhengduo Yang, Xipeng Zhang

**Affiliations:** ^1^Department of Pathology, Tianjin Union Medical Center, Tianjin 300121, China; ^2^Department of Colorectal Surgery, Tianjin Union Medical Center, Tianjin 300121, China

## Abstract

We previously reported that polyploid giant cancer cells (PGCGs) induced by CoCl_2_ could form through endoreduplication or cell fusion. A single PGCC formed tumors in immunodeficient mice. PGCCs are also the key contributors to the cellular atypia and associate with the malignant grade of tumors. PGCCs have the properties of cancer stem cells and produce daughter cells via asymmetric cell division. Compared with diploid cancer cells, these daughter cells express less epithelial markers and acquire mesenchymal phenotype with importance in cancer development and progression. Tumor budding is generally recognized to correlate with a high recurrence rate, lymph node metastasis, chemoresistance, and poor prognosis of colorectal cancers (CRCs) and is a good indicator to predict the metastasis and aggressiveness in CRCs. Micropapillary pattern is a special morphologic pattern and also associates with tumor metastasis and poor prognosis. There are similar morphologic features and molecular phenotypes among tumor budding, micropapillary carcinoma pattern, and PGCCs with their budding daughter cells and all of them show strong ability of tumor invasion and migration. In this review, we discuss the cancer stem cell properties of PGCCs, the molecular mechanisms of their regulation, and the relationships with tumor budding and micropapillary pattern in CRCs.

## 1. Introduction

Colorectal cancer (CRC) is one of the most common malignant tumors and its incidence ranks the third of malignant tumors [1]. The metastasis and relapse of CRC are the main reasons of tumor recurrence and patient death [2]. Twenty percent of CRCs have lymph node and/or distant metastasis at diagnosis [3]. The overall 5-year survival rate for CRCs patients is 64% and this rate drops to 12% in metastatic CRC patients [1]. The high death rate of metastatic CRCs is well known. If we can identify characteristic features in the primary lesion which are highly correlated to recurrence and metastasis of CRCs, then these characteristics can be used as prognostic markers to predict the recurrence or metastasis [4, 5]. The essential step in tumor invasion and metastasis is the tumor dedifferentiation and dissociation at the invasion front [6]. However, the degree of tumor differentiation in CRCs is hard to evaluate and is not exactly in accordance with the metastasis. Recently, increasing evidences confirmed the important role of polyploid giant cancer cells (PGCCs) and tumor budding in predicting the metastasis and patient's prognosis of CRCs [7, 8].

Normal human cells contain 46 chromosomes, but tumors cells contain abnormal numbers (usually between 60 and 90) of chromosomes, with cell-to-cell variability. Structural abnormalities of chromosomes such as inversions, deletions, duplications, and translocations are commonly observed in cancer cells but are rare in normal cells [9, 10]. The cells with abnormal number of chromosomes are named polyploid cells. PGCCs contribute to solid tumor heterogeneity and are the main histological feature of malignant tumor in pathologic diagnosis. Commonly, the number of PGCCs is higher in high-grade malignant tumor than in low-grade malignant tumor, in recurrent tumor after chemotherapy than in tumor before chemotherapy, and in the metastatic foci than in the primary tumor [11, 12]. PGCCs are previously considered to be at the stage of mitotic catastrophe and believed to be nondividing senescent cells. Polyploid giant cells appear in skeletal muscles, osteoclasts, and senescent cells [13] and can be formed via cell fusion or abortive cell cycles [14]. We previously found that PGCCs isolated from the ovarian cancer and breast cancer cell lines can revert to regular cancer cells through budding [12, 15]. PGCCs can express cancer stem cell markers including CD44 and CD133. The daughter cells budded from PGCCs expressed EMT-related proteins and show strong ability of tumor invasion and migration.

Tumor budding is similar to the morphological features of micropapillary pattern [16–20]. Based on the morphologic features, protein expression, and biologic behaviors, we speculate that these daughter cells budded from PGCCs fall into the broad term of tumor budding and micropapillary cancer pattern [12, 15, 21]. This review will discuss the recent development of PGCCs and its association with tumor budding and micropapillary pattern in CRCs.

## 2. PGCCs and Cancer Stem Cells

Cancer stem cells, often referred to as tumor-initiating or tumor-propagating cells [22, 23], are capable of generating entire tumor mass. These cells are considered as the seed cells to fuel the development, chemoresistance, and recurrence of human cancer. The history of the cancer stem cell can be traced to Coneheim who proposed the embryonic nest theory of cancer stem cells 150 years ago [24, 25]. The early definitive evidence of cancer stem cells was found in leukemias [26]. Later, Al-Hajj et al. and other groups showed that cancer stem cells were present in solid cancers, including breast carcinoma and glioblastoma [27, 28]. Intensive efforts have been devoted to identifying and characterizing cancer stem cells. To date, stem cell-like populations have been characterized and isolated by flow cytometry using so-called cancer stem cell markers [27–29]. However, these markers were neither pure nor specific for cancer stem cells [30–32]. Furthermore, the phenotypes of these marker-enriched cancer cells were not stable and could change from marker-positive to marker-negative [33]. Thus, characterizations of markers that unequivocally identify a population of cancer stem cells remain challenging. American Association for Cancer Research (AACR) consensus conference workshop described cancer stem cell as “a cell within a tumor that possesses the capacity to self-renew and to cause the heterogeneous lineages of cancer cells that comprise the tumor” [34].

PGCCs are often considered as the senescent cells. PGCCs in tumors have not attracted major attention due to lack of extensive study and their poorly understood biology [12]. Actually, PGCCs are the key contributors to cancer heterogeneity and form the basis for differential diagnosis of benign and malignant tumors. They associate with the malignant grade and lymph node metastasis. The relationship between PGCCs and cancer differentiation has long been known, but it is not clear if PGCCs contribute to tumorigenesis or they are only the consequence of malignant transformation [35–37]. Clinical evidence is accumulating in support of the view that the number of PGCCs positively correlates with the malignant degree of cancer. In cancer, multiple stresses including antimitotic chemotherapy drugs, radiotherapy, hypoxia, or poor microenvironment can increase the number of PGCCs.

We recently reported that PGCCs can be purified from human ovarian and breast cancer cells lines and primary human ovarian tumors with the use of chemical hypoxic mimetic, cobalt chloride (CoCl_2_) [12, 15, 21, 36–38], and confirmed that these cells were formed through cell fusion and produced daughter cells via asymmetric cell division. On the other hand, PGCCs are slow-cycling in nature and express stem cell markers. These cells are prone to differentiation into other benign tissues including adipose, cartilage, and bone, which has been confirmed both in vitro and in vivo [12]. A single PGCC can form spheroids in medium and Matrigel in vitro through time-lapse observation. A single PGCC can also form tumor when it was subcutaneously injected into the SCID mice. PGCCs express a distinct signature of proteins involved in hypoxia, invasion, chromatin remodeling, and cell cycle regulation [39–41]. These features of PGCCs suggest that PGCCs may represent a novel type of cancer stem cells which can be defined by size and morphology without using cell surface markers.

PGCCs generate daughter cells via asymmetric cell division which is a hallmark of stem cells. In multicellular eukaryotes, mitosis is the recognized process for somatic cell division, ensuring the accurate separation of genetic material [42, 43]. Asymmetric cell division is important in producing cell diversity during normal tissue development. In contrast to symmetric cell divisions, asymmetric cell division produces two daughter cells including stem cell and non-stem cell which have different cellular fates [44]. Asymmetric cell division is a fundamental process involving many physiological and pathological processes. In a typical outcome, the stem cell generates a copy of itself, and a second daughter cell programmed to differentiate into a non-stem cell type [45, 46]. Asymmetric division is a key mechanism ensuring tissue homeostasis, maintaining the stem and progenitor cell population, and allowing the development of diverse functional cells.

## 3. Tumor Budding and Its Clinical and Pathologic Significances

Prognosis of CRC patients has been associated with several morphological features including the infiltration depth, tumor cell differentiation, and lymph node or distant metastasis. These morphological features are the primary parameters for prognostic evaluation and tumor staging of CRCs in clinics [47]. However, other pathological indicators including tumor budding should be noted in the pathologic records to help the clinicians to judge the prognosis of patients with CRC [48]. Tumor budding is an increasingly recognizable feature to indicate the lymph node metastasis in CRCs [49–53]. “Tumor budding” was first described by Imai who noticed that cells sprouting from the edge of tumor entity are indicative of a tumor with high growth rate [54]. It is generally thought that tumor budding is a histological feature that is observed by pathologists from microscopy. The term “tumor budding” is referred to as a cluster of cancer cells which are located in the invasive front microscopically [55, 56]. Tumor budding is defined as an isolated single cancer cell or a cluster composed of fewer than five cancer cells and has been reported to be highly related to the recurrence rate and poor prognosis in CRCs [48, 49, 57–61]. Tumor cells with minimally differentiated CRCs show strong ability of tumor invasion and migration and lymph node metastasis. The number of tumor buddings has negative correlations with the degree of tumor differentiation. There are more tumor buddings in minimally differentiated CRCs than in the highly differentiated and moderately differentiated CRCs [57, 62–65]. In clinical practice, Dukes staging system has been widely used for CRC classification for many years because it can effectively predict the disease prognosis. However, some patients with the same Dukes stage have different prognosis and different response to chemotherapy. Comprehensive evaluation system including the degree and type of differentiation, tumor budding, lymph node metastasis, and infiltrative depth should be used to guide the clinical treatment and predict prognosis. The comprehensive evaluation system should be reproducible and have substantial predictive value for the patients with CRC.

Several large studies tried to establish the criteria of clinical significances of tumor budding in CRCs and confirmed that tumor budding correlates with the lymph node or distant metastasis and associates with patient prognosis. Hase et al. studied 663 patients with CRCs; they divided patients into two groups according to the number of buddings including none or mild group and moderate or severe group [55]. They found that the presence of moderate-to-severe tumor budding indicated a bad biological behavior of CRCs; they also proposed that meticulous follow-up and adjuvant chemotherapy may be beneficial to patients with moderate-to-severe tumor budding regardless of their Dukes staging. In a multivariate analysis, tumor budding but not diffuse infiltration was identified as an independent prognostic factor. Other studies showed that tumor budding is an independent factor to predict the prognosis of CRCs. Ueno et al. reported that the number of tumor buddings is associated with tumor metastasis in CRCs [66–69]. A count of 0 to 9 tumor buddings per field (magnification of ×250) was marked as low grade, and a count of 10 or more was considered as high grade [68]. Like the Gleason scores in cancers of the prostate, tumor budding may be one of the important scoring factors to predict the prognosis in CRC patients.

The morphologic characteristics of tumor budding reflect the loss of adhesive epithelial phenotype in cancer cells and are accompanied by the metastasis of cancer [70–73]. Results from Pyke et al. confirmed that laminin-5 is a marker of invading cancer cells because of similar distributions between laminin-5-positive budding cancer cells at the invasive front in CRCs and the receptor for urokinase-type plasminogen activator [74]. It was suggested that laminin-5 might represent a valuable marker for tumor budding. Furthermore, laminin-5 and urokinase-type plasminogen activator receptor colocalized in CRCs could be important in the invasion and metastasis of cancer cells [74].

## 4. The Role of EMT in the Process of Tumor Budding and PGCCs with Daughter Cells

EMT is involved in many physiological processes including mesoderm formation and neural tube formation and pathological processes including wound healing and organ fibrosis [75, 76]. It should be pointed out that EMT plays an essential role in cancer metastasis and progression as epithelial cells lose their cell polarity and cell-cell adhesion and become mesenchymal cells with migratory and invasive properties [77]. During EMT, cells lose their epithelial morphology because of the cytoskeleton reorganization. Low cell-cell adhesion resulted from E-cadherin dysfunction and different expression of tight and adherent junction proteins increased invasive properties of cancer cells [78].

EMT has also been demonstrated to play an essential role during the formation of cancer stem cells [79]. Previously, we confirmed that PGCCs can be induced by CoCl_2_ and paclitaxel. When cancer cell lines recovered from the treatment of CoCl_2_ and paclitaxel, PGCCs could produce daughter cells via asymmetric cell division. Western blot analysis was performed to confirm that cytokeratin (AE1/AE3) expression was lower in the daughter cells than in cancer cells without treatment. The increased expression of mesenchymal markers such as vimentin was evident in daughter cells [12, 15, 21]. Particularly after paclitaxel treatment, the daughter cells developed an elongated, spindle cell, fibroblastic morphology, which was consistent with mesenchymal cells [15]. A high-throughput iTRAQ-based proteomic methodology was used to determine the differentially expressed proteins between PGCCs treated with CoCl_2_ and the control cells. Results showed that a panel of stem cell-regulating factors and EMT-related proteins were upregulated in PGCCs [21]. PGCCs with budding daughter cells had higher Snail, TWIST, and Slug expression than the diploid control tumor cells [21].

## 5. Wnt/***β***-Catenin Signal Pathway in Tumor Budding

Jass et al. reported that APC (adenomatous polyposis coli) mutation was much less frequent in sporadic microsatellite instability-high (MSI-H) cancers than in MSI-low or microsatellite stable cancers [7]. APC can regulate the expression of *β*-catenin and is an important component of *β*-catenin degradation complex. Tumor budding was characterized by increased expression for both *β*-catenin and p16 (cyclin-dependent kinase inhibitor 2A) [68]. *β*-Catenin is important for linkage of E-cadherins to the cytoskeleton [80, 81]. The downregulation of E-cadherin in carcinoma cells is associated with increased invasive ability of cancer cells [82, 83]. Furthermore, epithelial cell adhesion molecule (Ep-CAM) has been confirmed to be involved in tumor budding [84]. Loss of membranous Ep-CAM regulates the *β*-catenin subcellular localization. When *β*-catenin translocates from the cytoplasm to the nucleus, epithelial adhesion was reduced and migratory potential increased. Loss of Ep-CAM of CRC cell membrane is highly correlated with tumor budding, cancer grade, and local recurrence [84].

## 6. Tumor Budding, Micropapillary Pattern, and PGCCs in CRCs

Micropapillary pattern was first reported in breast cancer, in which a small cluster of tumor cells appear in tumor tissue and there are no vessels and stromal cells in the middle of tumor cluster [16]. Micropapillary pattern has been detected in about 20% of CRCs, a phenomenon analogous to the more familiar one seen in some carcinomas of breast, bladder, lung, pancreas, ovary, urothelial tract, and stomach [85–88]. Micropapillary pattern in carcinomas is associated with a greater frequency of lymphovascular invasion and lymph node metastases and poor prognosis [89]. Tang et al. reported that the average number of metastatic lymph nodes, the proportion of CRC with distant metastasis, and the number of cases with lymphovascular tumor emboli were significantly higher in CRCs with micropapillary pattern compared to those in CRCs without micropapillary pattern. Furthermore, micropapillary pattern often appears in tumor with minimal differentiation and the expression of E-cadherin in tumor cells of micropapillary pattern is lower than that in regular tumor cells [89]. The difference between tumor budding and micropapillary pattern is mainly based on the location and cell number. Micropapillary pattern can appear in both the edge and center of tumor mass, and the cell number is often more than five. Tumor budding is located in the invasion front and the cell number is less than five. Verdú et al. reported that there is an “inside-out” MUC1 immunohistochemical staining feature in micropapillary pattern [90]. MUC1 plays an important role in the detachment of cells from the stroma and determines the characteristic morphological features of the invasive potential and aggressive behavior via regulating the intercellular adherence [91]. Micropapillary pattern has similar morphological features to tumor budding in the invasion front of CRCs [90].

PGCCs appear in most of micropapillary carcinoma patterns and tumor budding. In some micropapillary patterns, the absence of PGCCs may be due to the five-micrometer thickness for slide processing. According to the morphological observations in CRCs with large sample, we speculate that tumor budding and micropapillary pattern may have the same origin, which derive from PGCCs with their budding daughter cells. We previously reported that single PGCCs which appeared after paclitaxel treatment from an invasive breast cancer cell line MCF-7 formed cancer organotypic structure (COS) including glandular, vessel-like, and papilla-like structures in vitro [15]. The papilla-like structures derived from single PGCCs were made of PGCCs and their daughter cells. The morphology of the papilla-like structure resembled that of micropapillary pattern in human CRCs [15]. As described above, daughter cells budded from PGCCs in tumors have strong ability to invade and migrate. Both tumor budding and micropapillary pattern originated from PGCCs with budding daughter cells show strong invasive ability.

**Figure 1:**
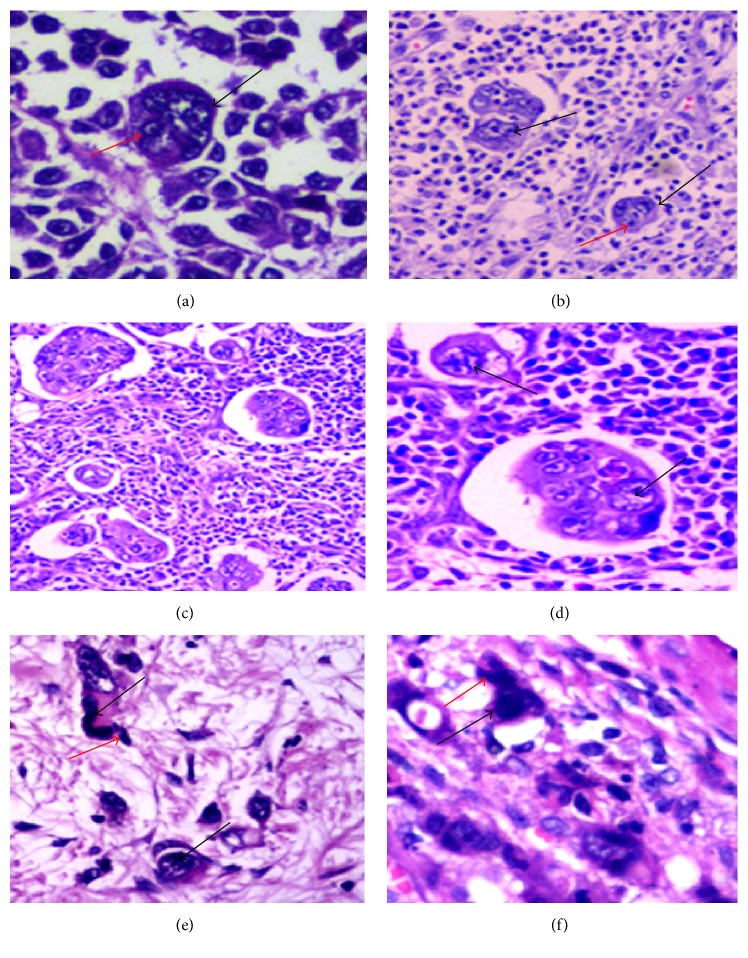
Tumor budding, PGCCs, and micropapillary carcinoma pattern in CRCs. ((a) and (b)) PGCCs with budding appear in minimally differentiated CRCs. Black arrows indicate PGCCs and red arrows indicate daughter cells generated by PGCCs (HE, ×200). (c) Micropapillary patterns appear in minimally differentiated CRC (HE, ×100). (d) PGCCs appear in micropapillary patterns (HE, ×100, black arrows). ((e) and (f)) Single PGCCs with their budding daughter cells in CRCs; and the structure is similar to tumor budding. Black arrows indicate PGCCs and red arrows indicate daughter cells budded by PGCCs (HE, ×200).

There is a close association among tumor differentiation, tumor budding, PGCCs, and micropapillary carcinoma pattern. Lv et al. also reported that single stromal PGCCs with their budding daughter cells were often associated with tumor metastasis in OSCs [92]. The number of single stromal PGCCs between low-grade and high-grade OSCs was different and statistically significant. High-grade OSCs have more single stromal PGCCs number [41, 92]. Single PGCCs generating daughter cells via budding can be observed in paraffin-embedded CRCs slides. Tumor tissue with single stromal PGCCs is more aggressive than tumor tissue without single stromal PGCCs. In another unpublished paper by us, we have confirmed that there are more tumor buddings and PGCCs in minimally differentiated CRCs than in highly differentiated and moderately differentiated CRCs. PGCCs can generate daughter cells via budding (Figures 1(a) and 1(b)). Because of the fact that the cell number in micropapillary pattern is more than that in tumor budding, there is a space separation between the tumor tissue and mesenchymal tissue. However, PGCCs always appear in micropapillary pattern (Figures 1(c) and 1(d)) and tumor budding (Figures 1(e) and 1(f)). Thus, the criterion of using single stoma PGCCs with their budding daughter cells may be better representative than tumor budding or micropapillary pattern. The association among tumor differentiation, tumor budding, PGCCs, and micropapillary pattern can help us further understand tumor metastasis and patient's prognosis.

## 7. Future Prospects of Tumor Budding and Single Stromal PGCCs

CRCs with tumor budding were identified to respond to chemoradiotherapy poorly and have adverse prognosis. Tumor budding is mostly unreported in daily diagnostic practice due to the lack of a standardized evaluating system. Karamitopoulou et al. suggested a method using 10 high-power fields to assess tumor budding at the invasive front. Their opinion showed that using 10 high-power fields to evaluate tumor budding had independent prognostic value and could show good interobserver consistency [93]. Jass et al. described “discrete clusters” of cells as buds [5]; Hase et al. gave definition of buds as clusters “appearing to bud from a larger gland” [55]. However, the identification of tumor budding is often confused with the fibroblasts, histiocytes masquerading, and fragmentation of a larger gland.

Since tumor budding is related to the prognosis of CRC patients, the standardized evaluating index including tumor budding and PGCCs could improve the grading of CRCs. Thus, it is worth paying more attention to the relationships between PGCCs with their newly budding daughter cells and tumor budding. The molecular mechanisms of tumor budding are involved in the expression of EMT-related proteins and Wnt/*β*-catenin signaling pathway which may provide clinicians with new hints to treat tumor with high-grade malignancy through targeting these proteins involved in tumor budding and PGCCs formation.

## References

[1] Siegel R., Naishadham D., Jemal A. (2013). Cancer statistics, 2013. *CA Cancer Journal for Clinicians*.

[2] Khan N., Mukhtar H. (2010). Cancer and metastasis: prevention and treatment by green tea. *Cancer and Metastasis Reviews*.

[3] Yoon S. S., Tanabe K. K. (1999). Surgical treatment and other regional treatments for colorectal cancer liver metastases. *The Oncologist*.

[4] Dukes C. E., Bussey H. J. R. (1958). The spread of rectal cancer and its effect on prognosis. *British Journal of Cancer*.

[5] Jass J. R., Love S. B., Northover J. M. A. (1987). A new prognostic classification of rectal cancer. *The Lancet*.

[6] Gabbert H. (1985). Mechanisms of tumor invasion: evidence from in vivo observations. *Cancer and Metastasis Review*.

[7] Jass J. R., Barker M., Fraser L. (2003). APC mutation and tumour budding in colorectal cancer. *Journal of Clinical Pathology*.

[8] Ricci-Vitiani L., Pallini R., Biffoni M. (2010). Tumour vascularization via endothelial differentiation of glioblastoma stem-like cells. *Nature*.

[9] Storchova Z., Kuffer C. (2008). The consequences of tetraploidy and aneuploidy. *Journal of Cell Science*.

[10] Storchova Z., Pellman D. (2004). From polyploidy to aneuploidy, genome instability and cancer. *Nature Reviews Molecular Cell Biology*.

[11] Zhang L., Ding P., Lv H. (2014). Number of polyploid giant cancer cells and expression of EZH2 are associated with VM formation and tumor grade in human ovarian tumor. *BioMed Research International*.

[12] Zhang S., Mercado-Uribe I., Xing Z., Sun B., Kuang J., Liu J. (2014). Generation of cancer stem-like cells through the formation of polyploid giant cancer cells. *Oncogene*.

[13] Walen K. H. (2006). Human diploid fibroblast cells in senescence; cycling through polyploidy to mitotic cells. *In Vitro Cellular and Developmental Biology—Animal*.

[14] Ogle B. M., Cascalho M., Platt J. L. (2005). Biological implications of cell fusion. *Nature Reviews Molecular Cell Biology*.

[15] Zhang S., Mercado-Uribe I., Liu J. (2014). Tumor stroma and differentiated cancer cells can be originated directly from polyploid giant cancer cells induced by paclitaxel. *International Journal of Cancer*.

[16] Vardar E., Yardim B. G., Vardar R., Ölmez M. (2015). Primary gastric invasive micropapillary carcinoma: a case report. *Turk Patoloji Dergisi*.

[17] Tajima S., Koda K. (2015). Transition between urothelial carcinoma in situ and non-invasive micropapillary carcinoma as a pivot connection between diverse morphologies of bladder carcinoma: a case report of urothelial carcinoma with villoglandular differentiation. *International Journal of Clinical and Experimental Pathology*.

[18] Cui Z.-Q., Feng J.-H., Zhao Y.-J. (2015). Clinicopathological features of invasive micropapillary carcinoma of the breast. *Oncology Letters*.

[19] Tanaka H., Baba Y., Sase T. (2014). Gastric intramucosal adenocarcinoma with an invasive micropapillary carcinoma component. *Clinical Journal of Gastroenterology*.

[20] Seo K.-J., Shin O. R., Lee J. Y., Choi Y.-J. (2015). Micropapillary urothelial carcinoma in a renal transplant recipient: a case report on urine cytomorphology emphasizing differentiation from high-grade urothelial carcinoma. *Cytopathology*.

[21] Zhang S., Mercado-Uribe I., Hanash S., Liu J. (2013). iTRAQ-based proteomic analysis of polyploid giant cancer cells and budding progeny cells reveals several distinct pathways for ovarian cancer development. *PLoS ONE*.

[22] Silván U., Díez-Torre A., Arluzea J., Andrade R., Silió M., Aréchaga J. (2009). Hypoxia and pluripotency in embryonic and embryonal carcinoma stem cell biology. *Differentiation*.

[23] Bonnet D., Dick J. E. (1997). Human acute myeloid leukemia is organized as a hierarchy that originates from a primitive hematopoietic cell. *Nature Medicine*.

[24] Heddleston J. M., Li Z., McLendon R. E., Hjelmeland A. B., Rich J. N. (2009). The hypoxic microenvironment maintains glioblastoma stem cells and promotes reprogramming towards a cancer stem cell phenotype. *Cell Cycle*.

[25] Virchow R. (1898). The Huxley lecture on recent advances in science and their bearing on medicine and surgery: delivered at the opening of the Charing Cross Hospital Medical School on October 3rd. *BMJ*.

[26] Lapidot T., Sirard C., Vormoor J. (1994). A cell initiating human acute myeloid leukaemia after transplantation into SCID mice. *Nature*.

[27] Al-Hajj M., Wicha M. S., Benito-Hernandez A., Morrison S. J., Clarke M. F. (2003). Prospective identification of tumorigenic breast cancer cells. *Proceedings of the National Academy of Sciences of the United States of America*.

[28] Hjelmeland A. B., Lathia J. D., Sathornsumetee S., Rich J. N. (2011). Twisted tango: brain tumor neurovascular interactions. *Nature Neuroscience*.

[29] Visvader J. E., Lindeman G. J. (2008). Cancer stem cells in solid tumours: accumulating evidence and unresolved questions. *Nature Reviews Cancer*.

[30] Gupta P. B., Chaffer C. L., Weinberg R. A. (2009). Cancer stem cells: mirage or reality?. *Nature Medicine*.

[31] Korkaya H., Wicha M. S. (2010). Cancer stem cells: nature versus nurture. *Nature Cell Biology*.

[32] Marotta L. L. C., Polyak K. (2009). Cancer stem cells: a model in the making. *Current Opinion in Genetics and Development*.

[33] Clevers H. (2011). The cancer stem cell: premises, promises and challenges. *Nature Medicine*.

[34] Clarke M. F., Dick J. E., Dirks P. B. (2006). Cancer stem cells—perspectives on current status and future directions: AACR workshop on cancer stem cells. *Cancer Research*.

[35] Donnelly N., Storchová Z. (2015). Causes and consequences of protein folding stress in aneuploid cells. *Cell Cycle*.

[36] Biancotti J.-C., Narwani K., Buehler N. (2010). Human embryonic stem cells as models for aneuploid chromosomal syndromes. *Stem Cells*.

[37] Thompson S. L., Compton D. A. (2010). Proliferation of aneuploid human cells is limited by a p53-dependent mechanism. *The Journal of Cell Biology*.

[38] Zhang S., Mercado-Uribe I., Sood A., Bast R. C., Liu J. (2016). Coevolution of neoplastic epithelial cells and multilineage stroma via polyploid giant cells during immortalization and transformation of mullerian epithelial cells. *Genes & Cancer*.

[39] Fei F., Zhang D., Yang Z. (2015). The number of polyploid giant cancer cells and epithelial-mesenchymal transition-related proteins are associated with invasion and metastasis in human breast cancer. *Journal of Experimental and Clinical Cancer Research*.

[40] Lopez-Sánchez L. M., Jimenez C., Valverde A. (2014). CoCl2, a mimic of hypoxia, induces formation of polyploid giant cells with stem characteristics in colon cancer. *PLoS ONE*.

[41] Qu Y., Zhang L., Rong Z., He T., Zhang S. (2013). Number of glioma polyploid giant cancer cells (PGCCs) associated with vasculogenic mimicry formation and tumor grade in human glioma. *Journal of Experimental and Clinical Cancer Research*.

[42] Bi E., Park H.-O. (2012). Cell polarization and cytokinesis in budding yeast. *Genetics*.

[43] Hu C.-K., Coughlin M., Field C. M., Mitchison T. J. (2008). Cell polarization during monopolar cytokinesis. *Journal of Cell Biology*.

[44] Zhang D., Wang Y., Zhang S. (2014). Asymmetric cell division in polyploid giant cancer cells and low eukaryotic cells. *BioMed Research International*.

[45] Shahriyari L., Komarova N. L. (2013). Symmetric vs. asymmetric stem cell divisions: an adaptation against cancer?. *PloS ONE*.

[46] Morrison S. J., Kimble J. (2006). Asymmetric and symmetric stem-cell divisions in development and cancer. *Nature*.

[47] Dukes C. E., Bussey H. J. (1958). The spread of rectal cancer and its effect on prognosis. *British Journal of Cancer*.

[48] Guzinska-Ustymowicz K. (2006). MMP-9 and cathepsin B expression in tumor budding as an indicator of a more aggressive phenotype of colorectal cancer (CRC). *Anticancer Research*.

[49] Manjula B. V., Augustine S., Selvam S., Mohan A. M. (2014). Prognostic and predictive factors in gingivo buccal complex squamous cell carcinoma: role of tumor budding and pattern of invasion. *Indian Journal of Otolaryngology and Head and Neck Surgery*.

[50] Satoh K., Nimura S., Aoki M. (2014). Tumor budding in colorectal carcinoma assessed by cytokeratin immunostaining and budding areas: possible involvement of c-Met. *Cancer Science*.

[51] Koelzer V. H., Langer R., Zlobec I., Lugli A. (2014). Tumor budding in upper gastrointestinal carcinomas. *Frontiers in Oncology*.

[52] Xie N., Wang C., Liu X. (2015). Tumor budding correlates with occult cervical lymph node metastasis and poor prognosis in clinical early-stage tongue squamous cell carcinoma. *Journal of Oral Pathology and Medicine*.

[53] Landau M. S., Hastings S. M., Foxwell T. J., Luketich J. D., Nason K. S., Davison J. M. (2014). Tumor budding is associated with an increased risk of lymph node metastasis and poor prognosis in superficial esophageal adenocarcinoma. *Modern Pathology*.

[54] Imai T. (1960). Growth patterns in human carcinoma. Their classification and relation to prognosis. *Obstetrics and Gynecology*.

[55] Hase K., Shatney C., Johnson D., Trollope M., Vierra M. (1993). Prognostic value of tumor ‘budding’ in patients with colorectal cancer. *Diseases of the Colon & Rectum*.

[56] Ha S. S., Choi H. J., Park K. J. (2005). Intensity of tumor budding as an index for the malignant potential in invasive rectal carcinoma. *Cancer Research and Treatment*.

[57] Koelzer V. H., Zlobec I., Lugli A. (2016). Tumor budding in colorectal cancer-ready for diagnostic practice?. *Human Pathology*.

[58] Graham R. P., Vierkant R. A., Tillmans L. S. (2015). Tumor budding in colorectal carcinoma: confirmation of prognostic significance and histologic cutoff in a population-based cohort. *American Journal of Surgical Pathology*.

[59] Koelzer V. H., Zlobec I., Berger M. D. (2015). Tumor budding in colorectal cancer revisited: results of a multicenter interobserver study. *Virchows Archiv*.

[60] O'Connor K., Li-Chang H. H., Kalloger S. E. (2015). Tumor budding is an independent adverse prognostic factor in pancreatic ductal adenocarcinoma. *American Journal of Surgical Pathology*.

[61] Angadi P. V., Patil P. V., Hallikeri K., Mallapur M. D., Hallikerimath S., Kale A. D. (2015). Tumor budding is an independent prognostic factor for prediction of lymph node metastasis in oral squamous cell carcinoma. *International Journal of Surgical Pathology*.

[62] Fukumoto K., Kikuchi E., Mikami S. (2016). Tumor budding, a novel prognostic indicator for predicting stage progression in T1 bladder cancers. *Cancer Science*.

[63] Grigore A., Jolly M., Jia D., Farach-Carson M., Levine H. (2016). Tumor Budding: the Name is EMT. Partial EMT. *Journal of Clinical Medicine*.

[64] Okamura T., Shimada Y., Nogami H. (2016). Tumor budding detection by immunohistochemical staining is not superior to hematoxylin and eosin staining for predicting lymph node metastasis in pT1 colorectal cancer. *Diseases of the Colon & Rectum*.

[65] Seki M., Sano T., Yokoo S., Oyama T. (2016). Histologic assessment of tumor budding in preoperative biopsies to predict nodal metastasis in squamous cell carcinoma of the tongue and floor of the mouth. *Head and Neck*.

[66] Ueno H., Mochizuki H., Hashiguchi Y. (2004). Risk factors for an adverse outcome in early invasive colorectal carcinoma. *Gastroenterology*.

[67] Ueno H., Mochizuki H., Hatsuse K., Hase K., Yamamoto T. (2000). Indicators for treatment strategies of colorectal liver metastases. *Annals of Surgery*.

[68] Ueno H., Murphy J., Jass J. R., Mochizuki H., Talbot I. C. (2002). Tumour ‘budding’ as an index to estimate the potential of aggressiveness in rectal cancer. *Histopathology*.

[69] Ueno H., Price A. B., Wilkinson K. H., Jass J. R., Mochizuki H., Talbot I. C. (2004). A new prognostic staging system for rectal cancer. *Annals of Surgery*.

[70] Karamitopoulou E. (2013). Tumor budding cells, cancer stem cells and epithelial-mesenchymal transition-type cells in pancreatic cancer. *Frontiers in Oncology*.

[71] Horcic M., Koelzer V. H., Karamitopoulou E. (2013). Tumor budding score based on 10 high-power fields is a promising basis for a standardized prognostic scoring system in stage II colorectal cancer. *Human Pathology*.

[72] Oshiro R., Yamamoto H., Takahashi H. (2012). C4.4A is associated with tumor budding and epithelial-mesenchymal transition of colorectal cancer. *Cancer Science*.

[73] Sert Bektaş S., Inan Mamak G., Çiriş I. M., Bozkurt K. K., Kapucuoğlu N. (2012). Tumor budding in colorectal carcinomas. *Turk Patoloji Dergisi*.

[74] Pyke C., Salo S., Ralfkiaer E., Romer J., Dano K., Tryggvason K. (1995). Laminin-5 is a marker of invading cancer cells in some human carcinomas and is coexpressed with the receptor for urokinase plasminogen activator in budding cancer cells in colon adenocarcinomas. *Cancer Research*.

[75] Thiery J. P., Acloque H., Huang R. Y. J., Nieto M. A. (2009). Epithelial-mesenchymal transitions in development and disease. *Cell*.

[76] Nieto M. A. (2009). Epithelial-Mesenchymal Transitions in development and disease: old views and new perspectives. *International Journal of Developmental Biology*.

[77] Mani S. A., Guo W., Liao M.-J. (2008). The epithelial-mesenchymal transition generates cells with properties of stem cells. *Cell*.

[78] Shimamura K., Hirano S., McMahon A. P., Takeichi M. (1994). Wnt-1-dependent regulation of local E-cadherin and *α*N-catenin expression in the embryonic mouse brain. *Development*.

[79] Mari P.-O., Verbiest V., Sabbioneda S. (2010). Influence of the live cell DNA marker DRAQ5 on chromatin-associated processes. *DNA Repair*.

[80] Lin L.-C., Hsu S.-L., Wu C.-L., Hsueh C.-M. (2014). TGF*β* can stimulate the p38/*β*-catenin/PPAR*γ* signaling pathway to promote the EMT, invasion and migration of non-small cell lung cancer (H460 cells). *Clinical and Experimental Metastasis*.

[81] Bastos L. G. D. R., de Marcondes P. G., de-Freitas-Junior J. C. M. (2014). Progeny from irradiated colorectal cancer cells acquire an EMT-like phenotype and activate wnt/*β*-catenin pathway. *Journal of Cellular Biochemistry*.

[82] Scarpa E., Szabó A., Bibonne A., Theveneau E., Parsons M., Mayor R. (2015). Cadherin switch during EMT in neural crest cells leads to contact inhibition of locomotion via repolarization of forces. *Developmental Cell*.

[83] Slusser A., Bathula C. S., Sens D. A. (2015). Cadherin expression, vectorial active transport, and metallothionein isoform 3 mediated EMT/MET responses in cultured primary and immortalized human proximal tubule cells. *PLoS ONE*.

[84] Gosens M. J. E. M., van Kempen L. C. L., van De Velde C. J. H., van Krieken J. H. J. M., Nagtegaal I. D. (2007). Loss of membranous Ep-CAM in budding colorectal carcinoma cells. *Modern Pathology*.

[85] Yang Y. L., Liu B. B., Zhang X., Fu L. (2016). Invasive micropapillary carcinoma of the breast: an update. *Archives of Pathology & Laboratory Medicine*.

[86] Bertz S., Wach S., Taubert H. (2016). Micropapillary morphology is an indicator of poor prognosis in patients with urothelial carcinoma treated with transurethral resection and radiochemotherapy. *Virchows Archiv*.

[87] Cao Y., Zhu L.-Z., Jiang M.-J., Yuan Y. (2016). Clinical impacts of a micropapillary pattern in lung adenocarcinoma: a review. *OncoTargets and Therapy*.

[88] Badyal R. K., Bal A., Das A., Singh G. (2016). Invasive micropapillary carcinoma of the breast: immunophenotypic analysis and role of cell adhesion molecules (CD44 and E-Cadherin) in nodal metastasis. *Applied Immunohistochemistry & Molecular Morphology*.

[89] Tang T., Zhang X.-J., Zhao L.-Z., Cao Y.-J., Xiao L., Wang R.-L. (2013). Relationship between colorectal adenocarcinoma with invasive micropapillary carcinoma component and lymph node metastasis. *Zhonghua Binglixue Zazhi*.

[90] Verdú M., Román R., Calvo M. (2011). Clinicopathological and molecular characterization of colorectal micropapillary carcinoma. *Modern Pathology*.

[91] Nassar H., Pansare V., Zhang H. (2004). Pathogenesis of invasive micropapillary carcinoma: role of MUC1 glycoprotein. *Modern Pathology*.

[92] Lv H., Shi Y., Zhang L. (2014). Polyploid giant cancer cells with budding and the expression of cyclin E, S-phase kinase-associated protein 2, stathmin associated with the grading and metastasis in serous ovarian tumor. *BMC Cancer*.

[93] Karamitopoulou E., Zlobec I., Kölzer V. (2013). Proposal for a 10-high-power-fields scoring method for the assessment of tumor budding in colorectal cancer. *Modern Pathology*.

